# Paraoxonase 3 Activity and The Ratio of Antioxidant to Peroxidation in The Follicular Fluid of Infertile Women

**Published:** 2014-03-09

**Authors:** Mohammad-Reza Rashidi Rashidi, Jalal Eisa-Khaje, Laya Farzadi, Masoud Darabi, Alieh Gasemzadeh, Vahideh Shahnazi, Shabnam Fayezi Fayezi, Amir Mehdizadeh, Reza Haji Hosseini, Mohammad Nouri

**Affiliations:** 1 Research Center for Pharmaceutical Nanotechnology, Tabriz University of Medical Sciences, Tabriz, Iran; 2 Women’s Reproductive Health Research Center, Alzahra Hospital, Tabriz University of Medical Sciences, Tabriz, Iran; 3 Department of Biochemistry and Clinical Laboratories, School of Medicine, Tabriz University of Medical Sciences, Tabriz, Iran; 3 Students Research Committee, Infertility and Reproductive Health Research Center, Shahid Beheshti University of Medical Sciences, Tehran, Iran; 5 Liver and Gastrointestinal Disease Research Center, Tabriz University of Medical Sciences, Tabriz, Iran; 6 Department of Biology, Payame Noor University, Tehran, Iran; 7 Umbilical Stem Cell Research Center, Tabriz University of Medical Sciences, Tabriz, Iran

**Keywords:** Infertility, PON3, Follicular Fluid, Peroxidation, *In Vitro* Fertilization

## Abstract

**Background:**

Paraoxonase-3 (PON3), as a high density lipoprotein (HDL)-associated lactonase, is capable of preventing the oxidative modification of low density lipoprotein (LDL). PON3 activity in follicular fluid (FF) is three times more than its activity in serum. However, the detailed role of PON3 in women’s fertility remains unknown. The aim of this study was to investigate the correlation between PON3 activity in the FF of women undergoing assisted reproductive technique (ART), *in vitro* fertilization (IVF), or intra-cytoplasmic sperm injection (ICSI).

**Materials and Methods:**

This cross-sectional study consisted of 50 women from couples with male factor infertility (MFI) or with female factor infertility (FFI). The FF samples were obtained during the ART intervention. PON3 activity, HDL cholesterol (HDL C), total antioxidant status (TAS) and the level of malondialdehyde (MDA) were determined. The morphology of the embryo was determined using embryo cell number (ECN) and embryo fragmentation score (EFS). In addition, fertilization rate (FR) was used an oocyte fertilization index.

**Results:**

Of 50 women, 20 women belonged to FFI group and the remaining 30 women belonged to MFI group. PON3 activity in FF of women in FFI group was significantly lower (p<0.05) in comparison with corresponding value in MFI group. The value of PON3 activity/MDA in the FFI group was lower than that in MFI group. Moreover, MDA level in the FF of FFI group was significantly higher (p<0.05) than its concentration in MFI group. Meanwhile, no significant difference was found in HDL-C concentration and TAS of both groups. No significant correlation was observed between the ECN and FF biochemical parameters. There was also a negative correlation between FR and MDA (r=-0.42, p=0.02), whereas a positive relation between FR with PON3 activity (r=0.59, p=0.004), HDL-C (r=0.35, p=0.04) and PON3/MDA (r=0.59, p=0.001).

**Conclusion:**

According to the results of this study, PON3 activity level as a key component of antioxidant system in FF may directly be associated with the success rate of ART and fertilization rate in women.

## Introduction

Free radicals cause oxidative damages to the cell membrane lipid content (1). The role of the reactive oxygen species (ROS) in peroxidation of lipids and their interference with sperm function, ovum function, and human reproduction have been reported (2). Natural byproduct of metabolism is ROS which includes the superoxide anion (O_2_ • –) and the hydroxyl radical (OH). ROS can induce DNA fragmentation, protein oxidation, lipid per oxidation and cellular damage (3-5). Within a cell, ROS is neutralized by the antioxidants (6). Paraoxonase (PON) is one of the strong antioxidants in the serum and the follicular fluid (FF). PON1 and PON3, which are both associated in serum with high density lipoprotein (HDL) cholesterol (C), protect the serum lipids from oxidation, probably through their ability to hydrolyze specific oxidized lipids (7, 8). The *PON* gene family consists of three members of *PON1* gene, *PON2* gene and PON3 gene, encoding *PON* enzyme family (4). All three genes of *PON* have been preserved in mammals, and this fact indicates the important physiological role of this antioxidant enzyme (9). There is a higher PON enzymatic activity in FF compared with serum, which has been attributed to PON expression and secretion by granulosa cells in FF (10).

PON3 is synthesized in the liver and carried in the blood in association with HDL. PON3 is also able to prevent the oxidation of low density lipoproteins (LDL) (11). Plasma concentration of PON3 is 100 times less than that of PON1 (12, 13). In recent studies, the activity of this enzyme in FF has been reported (14) and is calculated to be three times more than its original concentration in serum (15). However, the role of PON3 in women’s fertility has not yet been fully studied. Considering the strong antioxidant property of PON3 and its high concentration in the FF, it is likely that this enzyme plays an important role in the oogenesis, eggs quality and fertilization. In the present study, PON3 activity and the ratio of antioxidant to peroxidation in the FF of women with male factor infertility (MFI) and with female factor infertility (FFI) were compared after ovarian stimulation, while their variation with respect to the number of oocytes, embryo cell number (ECN), embryo fragmentation score (EFS) and fertilization rate (FR) were statistically analyzed.

## Materials and Methods

### Study design and subjects

In this cross-sectional study, we gained the agreement of the Ethical Committee of Tabriz University of Medical Science, and all patients gave written informed consent. Fifty infertile couples referred to Tabriz Alzahra Women’s Hospital, Tabriz, Iran, for infertility treatment using assist- ed reproductive technique (ART) were selected within a three-month period. Out of 50 couples, 30 women with MFI were used as the control group (MFI group), and the remaining 20 women with FFI were used as the test group (FFI group).

Among selected patients, 60% of infertile partner were male and 40% were female. Lack of infections and husband with no smoking habit were defined as including criteria. The long protocol, gonadotropin-releasing hormone agonist (GnRHa) and human menopausal gonado- tropin (HMG) were used for all subjects as the treatment protocol for the stimulation of ovulation (16). On the 14^th^ day of the menstrual cycle, the follicles larger than 3 mm were punctured and the FF was extracted. After separating the oocytes from FF, the remaining was centrifuged and the supernatant was kept at -86˚C for further studies. The collected oocytes were incubated at 37˚C, 5% CO_2_ and 95% humidity for 4 hours, and then were used for *in vitro* fertilization (IVF). In couples with abnormal sperm parameters such as low sperm count, low sperm motility and morphological defects, intracytoplasmaic sperm injection (ICSI) was used for oocytes fertilization, otherwise IVF was performed. Identification of zygotes was carried out 18 hours after insemination through the appearance of two pronuclei (2PN). All embryos were recultured in standard culture medium (ISM1) at 37˚C, 5% CO_2_ and 95% humidity for 24 hours. On the second day of the culture, the morphology of the embryos was determined using ECN and EFS (17, 18). In addition, FR was used as an oocyte fertilization index. FR was calculated as the number of fertilized oocytes/ number of mature oocytes ×100.

### Determination of simvastatinase PON3 activity

PON3 has a unique ability to metabolize the lipophilic agents such as lovastatin and simvastatin (19). 

PON3 activity was determined through monitoring of the conversion of simvastatin (SV) to β,δ- dihydroxyacid simvastatin (SVA) using reverse phase-high performance liquid chromatography (RP-HPLC) method as described before (20) with slight modification as follows: SV (120 µM) was incubated with FF at 37˚C for 60 minutes, and the reaction was terminated by addition of acetonitrile. The sample was centrifuged at 6000 rpm for 6 minutes, and the amount of SVA in the supernatant was analyzed using a high-performance liquid chromatography (HPLC) system (Waters Associates, Norwich, Cheshire, UK) which consisted of a Waters 515 pump, Waters 717 plus Autosampler, Waters 2487, and Dual λ Absorbance Detector. The mobile phase was a mixture of 100 mM monopotassium phosphate (pH=4.5)/ acetonitrile (27/73 v/v). Separations were performed on a C18 column (Phenosphere-LUNA, 5 μM, 250×4.6 mm) with a C18 guard column (Perfectsil Target ODS-3, 5 μM, 10×4 mm). The effluent was monitored by ultraviolet (UV) detection at 239 nm at a flow rate of 1 ml/minute. 

### Determination of MDA levels in FF

MDA levels in FF were determined by the thiobarbituric acid (TBA) method and expressed as nmol MDA formed/mL FF (20). Briefly, 0.5 ml FF was shaken with 2.5 ml of 20% trichloroacetic acid (TCA) in a 10 ml centrifuge tube. Then, 1ml of 0.67% TBA was added to the mixture, shaken, and heated in a boiling water bath for 60 minutes and it was cooled rapidly. MDA content in the serum was spectrophotometerically determined at 532 nm. The calibration curve was plotted with 0.1 to 20 μmol/L tetraethoxypropane (TEP).

### Measurement of plasma total antioxidant status (TAS) in FF

Total antioxidant status (TAS) was measured in FF using a commercial kit (Randox Laboratories, France). The assay was performed by incubation of 2,2'-azino-di-(3-ethylbenzthiazoline sulphonate) (ABTS) with a peroxidase (methmyoglobin) and hydrogen peroxide to develop a relatively stable blue-green color, which subsequently measured at 600 nm (21). Trolox, a traditional standard for TAS measurement, was used to calculate Trolox molar equivalent (22).

### Determining the HDL-C levels

HDL-C level was measured after extraction of the particles from FF by means of phosphotungstic solution and centrifugation. The amount of cholesterol in the supernatant was determined spectrophotometrically by cholesterol oxidase (Pars Azmon, Iran). 

### Statistical analysis

All data were expressed as mean ± SD. The normality test showed that all data were normal or nearly normally distributed. Statistical comparisons were performed using t test. ANOVA was used to evaluate the differences between the means of more than two groups. A value of p<0.05 was considered statistically significant. Statistical analysis was carried out by Statistical Package for the Social Sciences (SPSS; version 16, SPSS Inc., Chicago, USA).

## Results

The general information of the population study is shown in table 1. In the test group, 68% of the patients had normal ovaries, 18% had polycystic ovaries and 14% had abnormal ovaries (small ovaries, ovaries which were operated upon, and no eggs were produced), 4% of the test subjects had abnormal uteruses (small uterus and endometriosis) and 30% suffered from abnormal menstrual cycles.

**Table 1:** General information of the studied women


Factors	Number (% )

**Male factor**	30 (60%)
**Female factor:**	20 (40%)
Ovary	
Normal	34 (68%)
PCOS	9 (18%)
Abnormal	7 (14%)
Uterus	
Normal	48 (96%)
Abnormal	2 (4%)
Menstrual cycle	
Normal	35 (70%)
Abnormal	15 (30%)


PCOS; Polycystic ovary syndrome.

PON3 activity in the FF of the MFI group was found to be significantly (p<0.001) higher than that in the women with FFI (4.7 ± 0.8 vs. 3.8 ± 0.7 µmol/min/ml). In contrast, the concentration of MDA, a lipid peroxidation indicator, in the FF of MFI group was 3.1 ± 1.4 nmol/ml compared to the value of 4.2 ± 1.7 nmol/ml measured in FF of women with FFI (p=0.024). Therefore, the ratio of antioxidant to peroxidation, which was evaluated as PON3/MDA value in the control group, was also higher than the corresponding value in the women with FFI. No statistically significant difference was found in the HDL-C level in the FF of both groups (Table 2). Although there was no significant difference in the TAS levels in the women with MFI and FFI, the ratio of PON3 to TAS in two groups were statistically different (p= 0.013).

Biochemical factors measured in the FF based on the number of oocytes are displayed in table 3. No significant difference was observed in the studied factors with respect to the number of oocytes. 

**Table 2:** Follicular fluid biochemical parameters in the studied population


Chromosomal polymorphic variations	MFI(Mean ± SD)	FFI(Mean ± SD)	P value

**PON3 (µM/min/ml)**	4.7 ± 0.8*	3.8 ± 0.7	<0.001
**HDL-C (mg/dl)**	21.6 ± 11.4	20.0 ± 6.1	0.556
**TAS (mM)**	1.6 ± 0.3	1.6 ± 0.2	0.465
**MDA (µM)**	3.1 ± 1.4	4.2 ± 1.7	0.024
**PON3/MDA**	1.8 ± 0.9	1.0 ± 0.6	0.002
**PON3/HDL-C**	0.4 ± 0.5	0.2 ± 0.1	0.097
**HDL-C/MDA**	7.1 ± 4.0	6.5 ± 4.7	0.633
**PON3/TAS**	3.0 ± 0.7	2.5 ± 0.5	0.013


MFI; Male factor infertility, FFI; Female factor infertility and TAS; Total antioxidant status.

**Table 3:** Follicular fluid biochemical parameters according to the oocyte numbers in studied women


Factors	Number of the oocytes (number of subject)			P value
	1-5 (n=12)	5-10 (n=24)	>10 (n=14)	

**PON3 (µM/min/ml)**	4.1 ± 1.2	4.3 ± 0.9	4.5 ± 0.8	0.550
**HDL-C (mg/dl)**	18.4 ± 3.6	20.0 ± 10.2	23.5 ± 10.2	0.379
**TAS (mM)**	1.5 ± 0.9	1.6 ± 0.2	1.7 ± 0.2	0.515
**MDA (µM)**	3.1 ± 0.9	3.8 ± 1.9	3.5 ± 1.6	0.566
**PON3/MDA**	1.4 ± 0.4	1.5 ± 1.0	1.6 ± 0.9	0.840
**PON3/HDL-C**	0.2 ± 0.1	0.2 ± 0.1	0.4 ± 0.6	0.356
**HDL-C/MDA**	7.2 ± 3.7	7.5 ± 5.2	6.4 ± 3.8	0.681
**PON3/TAS**	2.6 ± 0.9	2.7 ± 0.6	2.8 ± 0.8	0.791


TAS; Total antioxidant status. Values are expressed as mean ± SD.

Comparison of number of the oocytes (t=1.32, p=0.13), EFS (t=0.81, p=0.41), ECN (t=0.89, p=0.32) and FR (t=1.52, p=0.11) between women with MFI and FFI by t test showed no significant difference between IVF and ICSI techniques. The relations between FF biochemical parameters with EFS, ECN and FR were assessed using regression analysis and the results have been summarized in table 4. A significant negative correlation was found between PON3 activity (r=-0.65, p=0.02) and PON3/MDA (r=-0.63, p=0.001) with EFS, whereas there was a positive correlation between EFS and MDA (r=0.55, p=0.002) which indicates that an increase in antioxidant/peroxidation value is accompanied with an increase in the embryo quality. No significant correlation was found between ECN and FF biochemical parameters. A negative correlation between FR and MDA (r=- 0.42, p=0.02), while a positive relations between FR and PON3 activity (r=0.56, p=0.004), HDL- C (r=0.35, p=0.041) and PON3/MDA (r=0.59, p=0.001) could be indicative of the beneficial effects of antioxidant in the success of ART. The same pattern was also observed when correlations were examined separately for women with MFI and FFI.

**Table 4:** Correlation of follicular fluid biochemical factors with embryo quality and fertilization rate


	EFS	ECN	FR

**PON3 activity (µM/min/ml)**	-0.65 (0.02)	0.24 (0.140)	0.56 (0.004)
**HDL-C (mg/dl)**	-0.25 (0.310)	0.07 (0.821)	0.35 (0.041)
**TAS (mM)**	-0.16 (0.425)	0.18 (0.283)	0.14 (0.404)
**MDA (µM)**	0.55 (0.002)	-0.38 (0.029)	-0.42 (0.020)
**PON3/MDA**	-0.63 (0.001)	0.22 (0.215)	0.59 (0.001)
**PON3/HDL-C**	-0.17(0.406)	0.15 (0.314)	0.22 (0.214)
**HDL-C/MDA**	-0.20 (0.374)	0.09 (0.703)	0.25 (0.246)


EFS; Emberyo fragmentation score, ECN; Emberyo cell number, FR; Fertilization rate and TAS; Total antioxidant status. The numbers in brackets are p values.

## Discussion

It has been shown that the concentration of lipidic hydroperoxides and active substances of thiobarbituric acid in the FF is lower than serum in women who underwent IVF. This confirms the presence of a suitable antioxidant in oocyte’s environment prior to ovulation (23). The concentration of this enzyme in FF is much higher than its concentration in serum. The results of this study shows that PON3 activity in FFI is significantly lower in comparison with the MFI group. According to a study by Closshey et al. (15) the level of PON3 activity in the FF in 14 infertile women who underwent IVF was higher in comparison with serum. Moreover, it was shown that there is a significant positive correlation between PON3 and the rate of laboratory pregnancy and fertility. These results match the results of this study. These findings along with high ratio of PON3/TAS in MFI group indicate the important role of PON3 in FF and in oogenesis.

PON3 is synthesized in the liver, attached to HDL and carried by the fluids in the body (11). It has been shown that HDL is the only lipoprotein which is present in FF (24). Therefore, it seems that HDL-C concentration in FF is associated with growth, oocyte maturation and rate of fertility in IVF (25). In the present study, no significant difference was found between the HDL-C levels of FF in MFI and FFI groups. Moreover, there was no significant association between HDL-C concentration and the number of oocytes. Contrary to the results of this study, Browne et al. have shown that the level of HDL-C of FF affects the number and quality of oocyte during stimulation of ovulation (26). It has been shown that older ages are associated with reduced amount of HDL apolipoprotein, which is accompanied by reduction of the number of mature oocytes in women (27). Although there are some reports verifying the role of HDL in oogenesis, further studies are needed on this issue. 

In our study, no significant difference was observed in the concentration of TAS in the two groups. In similar studies, no significant difference was found in the TAS levels in the women with FFI who suffered from endometriosis when compared with the TAS concentration of the MFI (28, 29). This is in line with the findings of this study. In present study, high level of PON3/TAS ratio in the control group (MFI group) and high level of MDA in FFI group indicate that PON3 plays an important role in the prevention of follicular oxidative stress. Furthermore, high level of MDA in FF of women with FFI could also be suggestive of the above-findings. This finding is similar to the findings of Yildrim et al. (30) who have shown that the lipidic peroxidation in the FF of the FFI group with polycyctic ovarian syndrome (PCOS) is much higher than that of the MFI group.

The results obtained in the present study show that the PON3/MDA ratio in the women with FFI is significantly lower than the corresponding value in the women with MFI (p=0.002). It could be stated that the ratio of antioxidant to peroxidation in the FF is a suitable factor for assessing the oxidative stress in the follicles.

PON3 is a strong antioxidant in FF. Closshey et al. (15) have reported that high PON3 activity inside follicle could probably be due to being produced locally in follicle. According to Browne et al. (26), the origin of the enzyme is granula-generating cells. In this study, for the first time, it was shown that PON3 activity in the FF of the women with FFI is lower than that in women with MFI. Now, it is known that PON3 is able to prevent LDL oxidation (10). This enzyme can utilize the products of lipids oxidation as substrates, and thus, reduces the severity of oxidative stress in the cell (31, 32). Therefore, it is more likely that PON3 have some important roles in the growth and maturation of the oocytes.

On the other hands, we were not able to find any significant difference between PON3 activity and the number of oocytes. Plachot et al. (33) have stated that the rate of fertilized oocytes is associated with not only the number but also the quality of oocytes. Thus, high level of PON3 activity during the growth period, maturity and quality of oocytes plays a vital role. Moreover, current study showed that the ratio of PON3 to MDA, as an indicator of antioxidant to peroxidation, in the FF of FFI group is lower than that of MFI group. Therefore, the antioxidant and peroxidation status in FF could be correlated with female infertility.

A significant negative relation between PON3 and PON3/MDA with EFS, and a positive relation between these parameters with FR may indicate that PON3 plays an important role in fertilization and the quality of embryo. A high level of PON3 in FF could be indicative of its specific role in development and maturation of good oocyte which, in turn, can lead to a healthy embryo.

The role of PON3 in fertility has not received enough attention. Browne et al. (34) have reported a significant negative association between HDL-C and EFS; however, they could not find any significant relation between EFS and PON3 activity in FF. Although the negative relation observed in our study between HDL-C and EFS was not statistically significant, we were able to show a significant positive relation between FR and HDL-C in FF. This may indicate the importance of HDL in fertilization which has also been reported by others (35). It has been shown that HDL and the proteins present in the structure of HDL could have a cytoprotective effects on oocyte and surrounding granulosa cells (36). As PON is one of the important antioxidant components of HDL and PON3 concentration in FF is much higher than its level in blood, it is likely that the local role of PON3 is much more dominant. It should be noted that studied variables including embryo quality and fertilization rate may be affected differentially in IVF and ICSI patients. However, because of our limited number of patients, it was not possible to perform two separate analyses for IVF and ICSI groups.

## Conclusion

Our findings confirm that PON3, as an antioxidant potential in follicular fluid, has a major role in regulating fertility and maintaining embryonic growth. Thus, PON3 could be a valuable therapeutic target to improve the success rate of ART.

## References

[1] Bezrukova GA (1991). Free radical oxidation of red blood cell membrane lipid structures as a trigger mechanism of an increase in red blood cell membrane permeability during blood coagulation *in vitro*. Gematol Transfuziol.

[2] Gupta S, Malhotra N, Sharma D, Chandra A, Ashok A (2009). Oxidative stress and its role in female infertility and assisted reproduction: clinical implications. Int J Fertil Steril.

[3] Cemeli E, Anderson D (2011). Mechanistic investigation of ROSinduced DNA damage by oestrogenic compounds in lymphocytes and sperm using the comet assay. Int J Mol Sci.

[4] Bedaiwy MA, Elnashar SA, Goldberg JM, Sharma R, Mascha EJ, Arrigain S (2012). Effect of follicular ﬂuid oxidative stress parameters on intracytoplasmic sperm injection outcome. Gynecol Endocrinol.

[5] Lopes AS, Lane M, Thompson JG (2010). Oxygen consumption and ROS production are increased at the time of fertilization and cell cleavage in bovine zygotes. Hum Reprod.

[6] Rizzo AM, Berselli P, Zava S, Montorfano G, Negroni M, Corsetto P (2011). Endogenous antioxidants and radical scavengers. Adv Exp Med Biol.

[7] Zhang C, Peng W, Wang M, Zhu J, Zang Y, Shi W (2010). Studies on protective effects of human paraoxonases 1 and 3 on atherosclerosis in apolipoprotein E knockout mice. Gene Ther.

[8] Browne RW, Shelly WB, Bloom MS, Ocque AJ, Sandler JR, Huddleston HG (2008). Distributions of high-density lipoprotein particle components in human follicular ﬂuid and sera and their associations with embryo morphology parameters during IVF. Hum Reprod.

[9] Primo-Parmo SL, Sorenson RC, Teiber J, La Du BN (1996). The human serum paraoxonase/arylesterase gene (PON1) is one member of a multigene family. Genomics.

[10] Aharoni A, Gaidukov L, Yagur S, Toker L, Silman I, Tawfik DS (2004). Directed evolution of mammalian paraoxonases PON1 and PON3 for bacterial expression and catalytic specialization. Proc Natl Acad Sci USA.

[11] Draganov DI, Stetson PL, Watson CE, Billecke SS, La Du BN (2000). Rabbit serum paraoxonase 3 (PON3) is a high density lipoprotein-associated lactonase and protects low density lipoprotein against oxidation. J Biol Chem.

[12] Draganov DI (2007). Human PON3, effects beyond the HDL: clues from human PON3 transgenic mice. Circ Res.

[13] Reddy ST, Wadleigh DJ, Grijalva V, Ng C, Hama S, Gangopadhyay A (2001). Human paraoxonase-3 is an HDL-associated enzyme with biological activity similar to paraoxonase-1 protein but is not regulated by oxidized lipids. Arterioscler Thromb Vasc Biol.

[14] Angelucci S, Ciavardelli D, Di Giuseppe F, Eleuterio E, Sulpizio M, Tiboni GM (2006). Proteome analysis of human follicular ﬂuid. Biochim Biophys Acta.

[15] Closshey WB, Browne RW, Huddleston HG, Sandler JR, Schisterman EF, Fujimoto VY (2007). High activity of paraoxonase 3 (PON3), a known potent antioxidant, identified in follicular ﬂuid. Fertil Steril.

[16] Ata B, Yakin K, Balaban B, Urman B (2008). GnRH agonist protocol administration in the luteal phase in ICSI-ET cycles stimulated with the long GnRH agonist protocol: a randomized, controlled double blind study. Hum Reprod.

[17] Steer CV, Mills CL, Tan SL, Campbell S, Edwards RG (1992). The cumulative embryo score: a predictive embryo scoring technique to select the optimal number of embryos to transfer in an in-vitro fertilization and embryo transfer programme. Hum Reprod.

[18] Puissant F, Van Rysselberge M, Barlow P, Deweze J, Leroy F (1987). Embryo scoring as a prognostic tool in IVF treatment. Hum Reprod.

[19] Draganov DI, Tiber JF, Speelman A, Osava Y, Sunahara R, La Du BN (2005). Human paraoxonases (PON1, PON2 and PON3) are lactonase with overlapping and distinct substrate specifcities. J Lipid Res.

[20] Suchocka Z, Swotowaka j, Pacheeka J, Suchocki P (2006). RPHPLC determination of paraoxonase-3 activity in human serum. J Pharm Biomed Anal.

[21] Conti M, Morand PC, Levillain P, Lemonnier A (1991). Improved ﬂuorimetric determination of malondialdehyde. Clin Chem.

[22] Miller NJ, Rice-Evans C, Davies MJ (1993). A new method for measuring antioxidant activity. Biochem Soc Trans.

[23] Jowzik M, Wolczynski S, Jozwik M, Szamatowicz M (1999). Oxidative stress markers in preovulatory follicular ﬂuid in human. Molecular Human Reprod.

[24] Becker S, von Otte S, Robenek H, Diedrich K, Nofer JR (2011). Follicular ﬂuid high-density lipoprotein-associated sphingosine 1-phosphate (S1P) promotes human granulosa lutein cell migration via S1P receptor type 3 and small Gprotein RAC1. Biol Reprod.

[25] Mehdizadeh A, Rahimipour A, Farzadi L, Darabi M, Shahnazi V, Shaaker M, Vatankhah AM (2011). Correlation between the level of cholesteryl ester transfer protein in follicular ﬂuid with fertilization rates in IVF/ ICSI cycles. Iran J Reprod Med.

[26] Browne RW, Shelly WB, Bloom MS, Ocque AJ, Sandler JR, Huddleston HG (2008). Distributions of high-density lipoprotein particle components in human follicular ﬂuid and sera and their associations with embryo morphology parameters during IVF. Hum Reprod.

[27] Von Wald T, Monisova Y, Hacker MR, Yoo SW, Penzias AS, Reindollar RR (2010). Age-related variations in follicular apolipoproteins may inﬂuence human oocyte maturation and fertility potential. Fertil Steril.

[28] Hong-Nerng Ho1, Ming-Yih Wu, Shee-Uan Chen, KuangHan Chao, Chin-Der Chen, Yu-Shih Yang (1997). Total antioxidant status and nitric oxide do not increase in peritoneal ﬂuids from women with endometriosis. Human Reproduction.

[29] Bedaiwy M, Agarwal A, Said TM, Goldberg JM, Sharma RK, Worley S (2006). Role of total antioxidant capacity in the differential growth of human embryos *in vitro*. Fertil Steril.

[30] Yildirim B, Demir S, Temur I, Erdemir R, Kaleli B (2007). Lipid peroxidation in follicular ﬂuid of women with polycystic ovary syndrome during assisted reproduction cycles. J Reprod Med.

[31] Aviram M, Kaplan M, Rosenblat M, Fuhrman B (2005). Dietary antioxidants and paraoxonases against LDL oxidation and atherosclerosis development. Handb Exp Pharmacol.

[32] Draganov DI, Stetson PL, Watson CE, Billecke SS, La Du BN (2000). Rabbit serum paraoxonase 3 (PON3) is a high density lipoprotein-associated lactonase and protects low density lipoprotein against oxidation. J Biol Chem.

[33] Plachot M, Belaisch-Allart J, Chouraqui A, Tesquir A, Serkine AM, Agaheyrached F (2003). Oocyte and embryo quality in poly cystic ovary syndrome. Gynecol Obstet Fertil.

[34] Browne RW, Shelly WB, Bloom MS, Ocque AJ, Sandler JR, Huddleston HG (2008). Distributions of high-density lipoprotein particle components in human follicular ﬂuid and sera and their associations with embryo morphology parameters during IVF. Hum Reprod.

[35] Browne RW, Bloom MS, Shelly WB, Ocque AJ, Huddleston HG, Fujimoto VY (2009). Follicular ﬂuid high density lipoprotein-associated micronutrient levels are associated with embryo fragmentation during IVF. J Assist Reprod Genet.

[36] Becker S, von Otte S, Robenek H, Diedrich K, Nofer JR (2011). Follicular ﬂuid high-density lipoprotein-associated sphingosine 1-phosphate (S1P) promotes human granulosa lutein cell migration via S1P receptor type 3 and small Gprotein RAC1. Biol Reprod.

